# Identification of functional regulatory elements in the human genome using pooled CRISPR screens

**DOI:** 10.1186/s12864-020-6497-0

**Published:** 2020-01-31

**Authors:** Samantha M. Borys, Scott T. Younger

**Affiliations:** 1 Broad Institute of MIT and Harvard, Cambridge, MA 02142 USA; 2 Center for Pediatric Genomic Medicine, Children’s Mercy Kansas City, Kansas City, MO 64108 USA; 3 Children’s Mercy Research Institute, Children’s Mercy Kansas City, Kansas City, MO 64108 USA; 4 Department of Pediatrics, University of Kansas Medical Center, Kansas City, KS 66160 USA; 5 Department of Pediatrics, University of Missouri-Kansas City School of Medicine, Kansas City, MO 64110 USA

**Keywords:** CRISPR, CRISPR screen, Regulatory element, Enhancer, p53

## Abstract

**Background:**

Genome-scale pooled CRISPR screens are powerful tools for identifying genetic dependencies across varied cellular processes. The vast majority of CRISPR screens reported to date have focused exclusively on the perturbation of protein-coding gene function. However, protein-coding genes comprise < 2% of the sequence space in the human genome leaving a substantial portion of the genome uninterrogated. Noncoding regions of the genome harbor important regulatory elements (e.g. promoters, enhancers, silencers) that influence cellular processes but high-throughput methods for evaluating their essentiality have yet to be established.

**Results:**

Here, we describe a CRISPR-based screening approach that facilitates the functional profiling of thousands of noncoding regulatory elements in parallel. We selected the tumor suppressor p53 as a model system and designed a pooled CRISPR library targeting thousands of p53 binding sites throughout the genome. Following transduction into dCas9-KRAB-expressing cells we identified several regulatory elements that influence cell proliferation. Moreover, we uncovered multiple elements that are required for the p53-mediated DNA damage response. Surprisingly, many of these elements are located deep within intergenic regions of the genome that have no prior functional annotations.

**Conclusions:**

This work diversifies the applications for pooled CRISPR screens and provides a framework for future functional studies focused on noncoding regulatory elements.

## Background

The ability to modify genomic DNA using the CRISPR/Cas9 system has rapidly transformed the field of functional genomics [1–4]. In addition to its applications in high fidelity genome engineering, the CRISPR/Cas9 system can be readily adapted for use in lentiviral-based pooled genetic screens [5, 6]. Pooled CRISPR screens permit the rapid identification of genes involved in a wide variety of biological processes and have become a routine experimental approach for dissecting complex genetic pathways [7, 8]. Although commonly used for characterizing the impact of gene knockout on cellular phenotypes, advances in CRISPR-based methods have enabled pooled CRISPR screens that profile the consequences of gene activation or gene repression [9–14].

The majority of CRISPR screens reported to date have focused exclusively on the function of protein-coding genes. In contrast, relatively few reports have described pooled screens that interrogate the function of noncoding regulatory elements. Many of the studies that have utilized pooled screens to characterize regulatory elements have designed dense tiling CRISPR libraries with genomic target sites that are restricted to sequences immediately adjacent to a gene of interest [15–17]. Isolated reports have described pooled CRISPR screens that target regulatory elements dispersed throughout the genome. For example, a pooled CRISPR screen targeting 685 p53-bound regions was able to identify a functional enhancer element upstream of CDKN1A [18]. In addition, a pooled CRISPR screen targeting 398 AP1-bound regions was able to identify an enhancer element that regulates FOXF1 expression [19]. While these studies have provided proof of concept for the application of pooled CRISPR screening in the functional characterization of regulatory elements, they were focused on profiling predicted regulatory elements as opposed to the identification of novel regulatory elements. Furthermore, they were not designed to yield generalizable insights into screening methodologies. Importantly, the practical considerations for the design and execution of pooled CRISPR screens that profile the function of noncoding regulatory elements at genome scale have yet to be defined.

The tumor suppressor p53 is a master regulator of cell fate decisions and a central line of defense against genomic instability [20–24]. While traditionally considered a transcription factor that binds to gene promoters and regulates gene expression, several recent reports have found that p53 binds predominantly to putative enhancer elements [25–29]. Multiplexed reporter assays have further revealed that the majority of genomic sequences bound by p53 exhibit potent enhancer activity [30, 31]. Moreover, p53 has been shown to modulate chromatin accessibility at a subset of enhancer elements in response to DNA damage [30]. While these studies have suggested that enhancer regulation is an important component of the p53 network the functional significance of p53-bound regulatory elements in the context of cell fate decisions remains unclear.

Here, we use p53 as a model system to evaluate pooled CRISPR screening methods for characterizing the function of noncoding regulatory elements. We designed a pooled CRISPR library targeting p53 binding sites throughout the genome and profile the functional significance of these sites in multiple biological contexts. We demonstrate that pooled CRISPR screens are capable of distinguishing p53-bound regulatory elements that influence cell proliferation and/or cell cycle arrest in response to DNA damage. While some of the regulatory elements we identified are well-characterized p53 targets, many are located within intergenic regions of the genome that lack prior functional annotations. Importantly, orthogonal experimental approaches were able to confirm the functional significance for several of these intergenic regulatory elements.

In addition to identifying p53-bound regulatory elements that influence cell proliferation and/or cell cycle arrest in response to DNA damage we explore a variety of practical considerations for the use of pooled CRISPR screens to profile the function of regulatory elements. Most notably, we perform each of our screens using both CRISPR interference (CRISPRi) and CRISPR knockout (CRISPRko) technologies allowing us to directly compare the different screening approaches. Surprisingly, we observed minimal overlap in screening results across technologies and demonstrate that screens performed using CRISPRi more closely recapitulate known biology. Altogether, our findings provide valuable insight into the design of CRISPR-based screening approaches for profiling the function of noncoding regulatory elements.

## Results

### CRISPR-mediated knockout of wildtype p53 increases cell proliferation in a subset of cancer cell lines

**Fig. 1:**
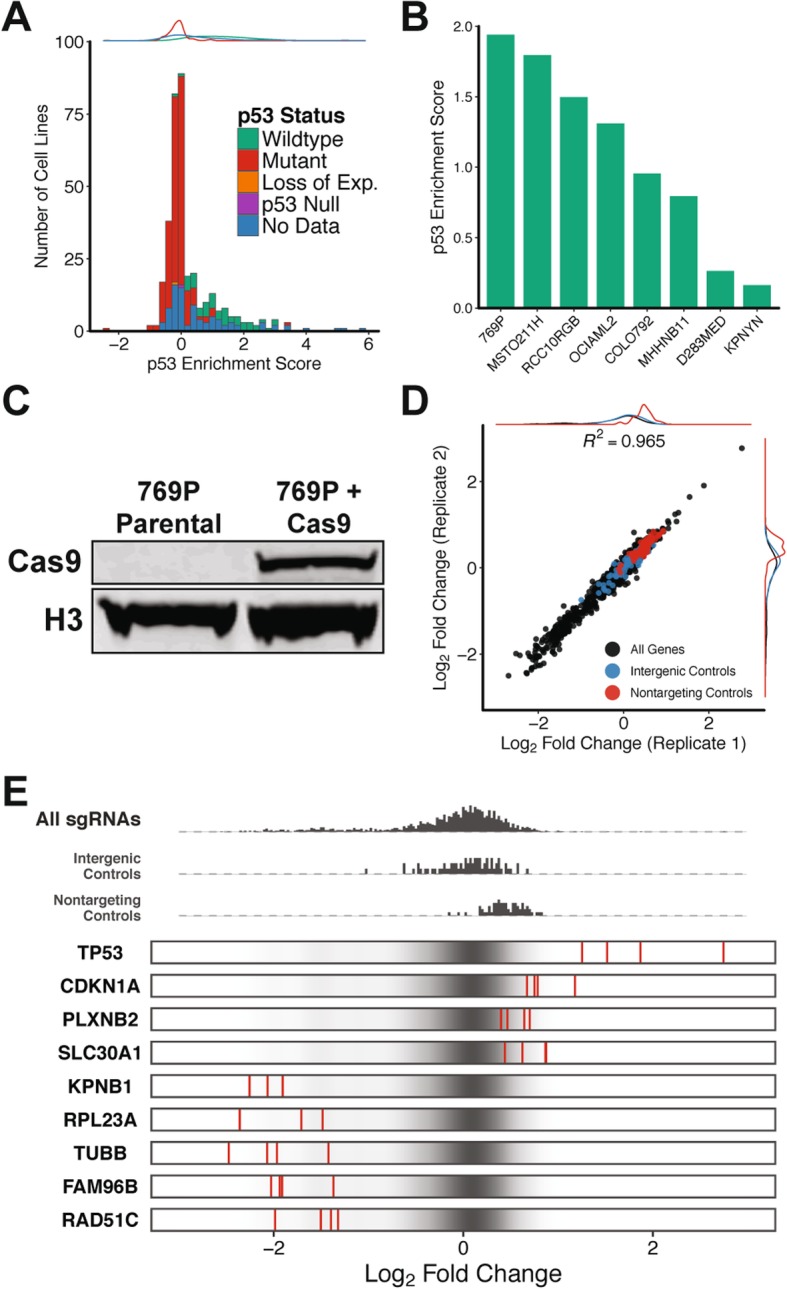
p53 knockout increases cell proliferation. **a** Distribution of p53 enrichment scores from pooled CRISPR knockout screens in 350 cancer cell lines. **b** p53 enrichment scores in a selected subset of cancer cell lines containing wildtype p53. **c** Western blot analysis of Cas9 expression in 769P cells. **d** Comparison of log_2_ fold changes (relative to pDNA) for all sgRNAs in CRISPR library between replicates. **e** Visualization of enrichment/depletion for sgRNAs targeting a selected subset of genes (red) compared to all sgRNAs in CRISPR library (black)

In order to identify an ideal cell-based model system to profile p53 function we took advantage of publicly available data generated through Project Achilles [32]. Briefly, Project Achilles utilizes genome-scale CRISPR knockout screens to identify genetic dependencies across a large compendium of cancer cell lines. The effect of knocking out each individual gene during a CRISPR screen is reported as a gene-level ‘Enrichment Score’. These scores are calculated based on changes in the relative abundance of cells harboring sgRNAs targeting each respective gene over the course of a screen. Therefore, these ‘Enrichment Scores’ serve as a proxy for the impact of gene knockout on cell proliferation. We profiled p53 ‘Enrichment Scores’ across more than 350 cancer cell lines and found that p53 knockout had no effect on cell proliferation for many of the cell lines screened in Project Achilles. However, we were able to identify a subset of cell lines in which p53 knockout conferred a proliferative advantage (Fig. 1a).

To identify molecular features associated with cell lines in which p53 knockout resulted in a proliferative advantage we intersected p53 ‘Enrichment Scores’ with data from the IARC (International Agency for Research on Cancer) TP53 database [33]. The IARC TP53 database is a curated resource for the mutation status of p53, along with several other known tumor suppressors and oncogenes, in human cell lines. Consistent with known p53 biology, we found that the proliferative advantage of p53 knockout was specific to cell lines harboring wildtype p53 (Fig. 1a, Additional file 1: Figure S1, Additional file 6: Table S1). In contrast, p53 knockout in cell lines containing mutations in the p53 gene, loss of p53 expression mutations, or p53 deletions had no significant impact on cell proliferation (Fig. 1a, Additional file 1: Figure S1, Additional file 6: Table S1). Collectively, these results indicate that cell proliferation can be used as a phenotype to screen p53 function in cell lines harboring wildtype copies of the gene.

To select a cell line for screening p53 function we first narrowed the list of cancer cell lines screened through Project Achilles down to those harboring wildtype p53. We then used data from the IARC TP53 database to further restrict this list to cell lines with no documented mutations in other known tumor suppressors or oncogenes (e.g. PTEN, KRAS, BRAF) (Additional file 6: Table S1). In total, we identified 8 cell lines that met our stringent criteria (Fig. 1b). The human renal adenocarcinoma cell line 769P displayed the highest p53 ‘Enrichment Score’ in the Project Achilles data and was selected as a model cell line for all subsequent experiments (Fig. 1b).

### Pooled CRISPR screen identifies p53-regulated genes that influence cell proliferation

To determine if a pooled CRISPR screen would be able to identify downstream targets of p53 that influence cell proliferation we designed a proliferation-based CRISPR screen. We generated a list of 330 genes that have p53 binding sites within 10 kb of their transcription start site and have been predicted to be directly regulated by p53 in a previous study [29]. We constructed a CRISPR library containing 4 sgRNAs targeting each gene in this list as well as 4 sgRNAs targeting p53 (Additional file 7: Table S2). As controls this CRISPR library included 70 sgRNAs targeting intergenic regions of the human genome and 70 sgRNAs with no genomic targets (Additional file 7: Table S2). We refer to this library throughout this report as our gene-targeting CRISPR library.

In order to perform CRISPR knockout (CRISPRko) screens we next generated a 769P-derived cell line expressing Cas9. We stably integrated Cas9 into a population of 769P cells using lentivirus and confirmed Cas9 expression by western blot (Fig. 1c). We then infected the Cas9-expressing 769P cells with our gene-targeting library at a multiplicity of infection (MOI) of ~ 0.5 and a representation of 1000 cells per sgRNA. Library-infected cells were cultured for 21 days, genomic DNA was isolated, and targeted sequencing was performed to evaluate changes in sgRNA abundance relative to the CRISPR library pDNA (Additional file 8: Table S3).

To calculate changes in sgRNA abundance over the course of the screen we utilized MAGeCK, a computational tool for model-based analysis of pooled CRISPR screens [34]. Analysis with MAGeCK revealed a significant correlation in sgRNA enrichment/depletion across biological replicates indicating that our screening results are highly reproducible (Fig. 1d, Additional file 9: Table S4). Moreover, sgRNAs targeting p53 were among the most enriched in our screen, confirming the validity of our approach (Fig. 1e, Additional file 10: Table S5). In addition to p53 we identified several p53-regulated genes in which knockout resulted in a significant proliferative advantage (Fig. 1e, Additional file 10: Table S5). Interestingly, we also uncovered a subset of p53-regulated genes where knockout lead to a proliferative disadvantage (Fig. 1e, Additional file 10: Table S5). These data demonstrate that proliferation-based CRISPR screens can be used to functionally profile downstream events in the p53 pathway.

### Pooled CRISPR screen identifies p53-bound regulatory elements that influence cell proliferation

**Fig. 2:**
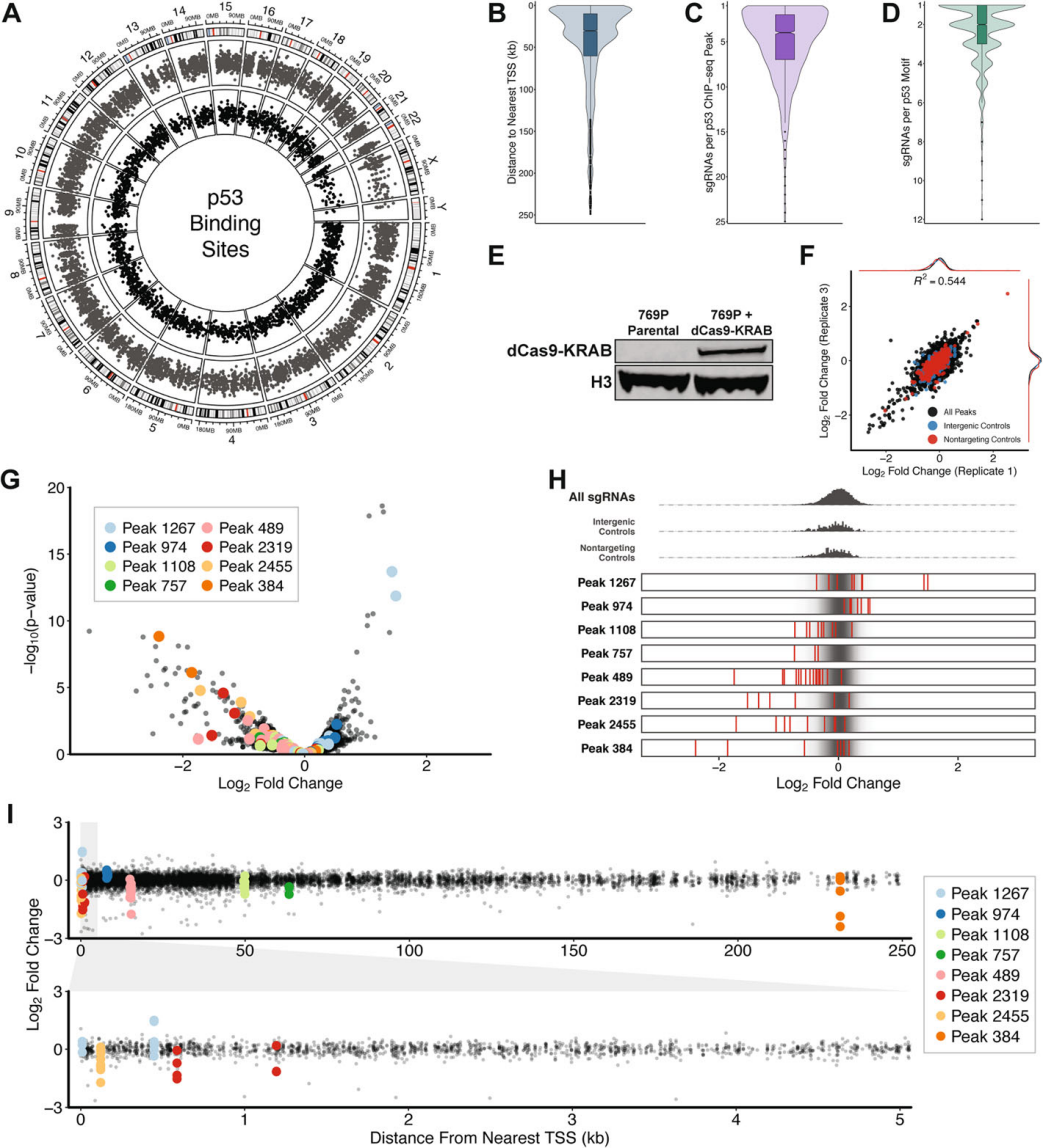
p53-bound regulatory elements influence cell proliferation. **a** p53 binding sites as determined by ChIP-Seq (black) and p53 consensus motifs (grey). **b** Distribution of distances to nearest annotated transcription start site for all sgRNAs in CRISPR library. **c** Distribution of number of sgRNA designs per p53 ChIP-Seq peak. **d** Distribution of number of sgRNA designs per p53 consensus motif. **e** Western blot analysis of dCas9-KRAB expression in 769P cells. **f** Comparison of log_2_ fold changes (relative to pDNA) for all sgRNAs in CRISPR library between replicates. **g** Volcano plot comparing significance of sgRNA enrichment/depletion and log_2_ fold change (relative to pDNA) for all sgRNAs in CRISPR library. **h** Visualization of enrichment/depletion for sgRNAs targeting a selected subset of peaks (red) compared to all sgRNAs in CRISPR library (black). **i** Comparison of log_2_ fold change (relative to pDNA) and distance from nearest annotated TSS for all sgRNAs in CRISPR library

Having established that CRISPR screens can be used to profile downstream events in the p53 pathway we next designed a screening approach to identify regulatory elements bound by p53 that mediate its influence on cell proliferation. More specifically, we designed a CRISPR library to target and inhibit the function of p53-bound regulatory elements. We used previously reported p53 ChIP-Seq data to identify p53 binding sites throughout the human genome [29]. We then searched for p53 consensus motifs (CWWG [N]_2-12_CWWG) located within each p53 ChIP-Seq peak (Fig. 2a). Once found, we designed sgRNAs targeting all PAM-containing sequences located within 16 bp upstream or downstream of the consensus motif. In total, we designed 11,434 sgRNAs targeting 4930 motifs located within 2036 p53 ChIP-Seq peaks (Fig. 2b, c, d, Additional file 11: Table S6). While many p53 motifs could only be targeted by a single sgRNA, the majority of the motifs we identified were targeted by multiple sgRNAs in our CRISPR library (Fig. 2d). Likewise, 83% (1703/2036) of the ChIP-Seq peaks represented in our CRISPR library were targeted by multiple sgRNAs (Fig. 2c). As controls we also included 500 sgRNAs targeting intergenic regions of the human genome and 500 sgRNAs with no genomic targets (Additional file 11: Table S6). We refer to this library throughout this report as our peak-targeting CRISPR library.

In order to perform CRISPR interference (CRISPRi) screens we next generated a 769P-derived cell line expressing a nuclease-dead version of Cas9 fused to the KRAB repressive domain (dCas9-KRAB). We stably integrated dCas9-KRAB into a population of 769P cells using lentivirus and confirmed dCas9-KRAB expression by western blot (Fig. 2e). We then infected the dCas9-KRAB-expressing 769P cells with our peak-targeting library at an MOI of ~ 0.5 and a representation of 1000 cells per sgRNA. Library-infected cells were cultured for 21 days, genomic DNA was isolated, and targeted sequencing was performed to evaluate changes in sgRNA abundance relative to the CRISPR library pDNA (Additional file 12: Table S7).

We again used MAGeCK to calculate changes in sgRNA abundance during the screen and observed a moderate correlation in sgRNA enrichment/depletion across biological replicates (Fig. 2f, Additional file 13: Table S8). Among the most enriched sgRNAs in the screen were those targeting a ChIP-Seq peak (Peak 974) located upstream of CDKN1A, a gene that was significantly enriched in screens performed with the gene-targeting CRISPR library (Fig. 2g, Additional file 14: Table S9**)**. Surprisingly, we identified many p53 binding sites in which CRISPRi-mediated repression resulted in a significant proliferative disadvantage (Fig. 2g, h). While some of these p53 binding sites were located proximal to an annotated transcription start site (TSS), most were located more than 10 kb away from the nearest TSS (Fig. 2i). Collectively, these data demonstrate that proliferation-based CRISPRi screens can be used to functionally profile regulatory elements that are bound by p53.

To evaluate the ability of CRISPRko technology to identify functional regulatory elements we performed screens using our peak-targeting CRISPR library in cells expressing Cas9 as opposed to dCas9-KRAB. We infected Cas9-expressing 769P cells with our peak-targeting CRISPR library at an MOI of ~ 0.5 and a representation of 1000 cells per sgRNA, cultured the infected cells for 21 days, isolated genomic DNA, and performed targeted sequencing to evaluate changes in sgRNA abundance relative to the CRISPR library pDNA (Additional file 15: Table S10). Analysis with MAGeCK revealed a moderate correlation in sgRNA enrichment/depletion across biological replicates indicating that our screening results are reproducible (Additional file 2: Figure S2A, Additional file 16: Table S11). Similar to our findings in dCas9-KRAB-expressing 769P cells we identified many p53 binding sites in which CRISPR-mediated knockout resulted in a significant proliferative disadvantage (Additional file 2: Figure S2B, Additional file 2: Figure S2C, Additional file 17: Table S12). Once again, most of these p53 binding sites were located more than 10 kb away from the nearest TSS (Additional file 2: Figure S2D). Interestingly, we observed minimal overlap in the sgRNAs that were significantly enriched/depleted across the CRISPRko and CRISPRi screens. Moreover, the overall concordance of enrichment/depletion for all sgRNAs in the peak-targeting CRISPR library was strikingly low (Additional file 2: Figure S2E). In contrast to our CRISPRi screen results we were unable to associate any p53 binding sites identified in the CRISPRko screen with genes that were significantly enriched/depleted in our gene-targeting CRISPR screen. Based on these data we focused our validation efforts on p53 binding sites identified in our CRISPRi screen.

### Repression of p53-bound regulatory elements impacts cell proliferation

**Fig. 3:**
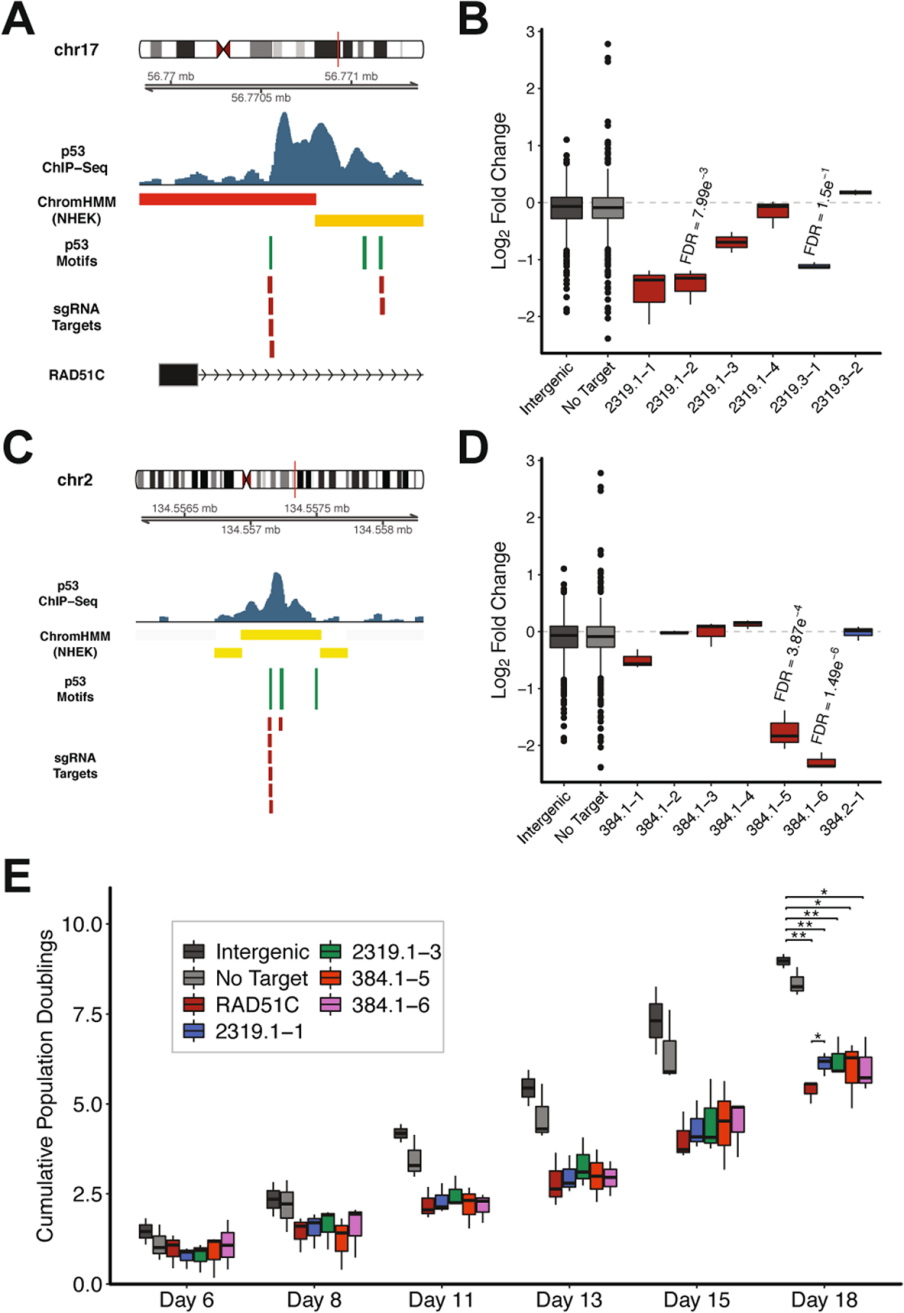
Functional characterization of p53-bound regulatory elements that influence cell proliferation. **a** Schematic of p53 motifs and sgRNA targets located in Peak 2319. (ChromHMM track legend: red = active promoter; orange = strong enhancer) (**b**) Log_2_ fold changes (relative to pDNA) in CRISPR screen for sgRNAs targeting Peak 2319. FDR values were calculated using the Benjamini-Hochberg method. **c** Schematic of p53 motifs and sgRNA targets located in Peak 384. (ChromHMM track legend: yellow = weak/poised enhancer) (**d**) Log_2_ fold changes (relative to pDNA) in CRISPR screen for sgRNAs targeting Peak 384. FDR values were calculated using the Benjamini-Hochberg method. **e** Comparison of cellular growth rates following inhibition of Peak 2319 or Peak 384. *P*-values were calculated using the two-tailed unpaired Student’s t-test with equal variances. **P < 0.01, *P < 0.05

Among the sgRNAs that were most depleted in our peak-targeting CRISPRi screen were those targeting Peak 2319 (Fig. 2h). Peak 2319 is located within the first intron of RAD51C, a gene determined to be essential for cell proliferation in our gene-targeting CRISPRko screen (Fig. 3a, Fig. 1e). Peak 2319 contains three p53 motifs, two of which were targeted by sgRNAs in our peak-targeting CRISPR library (Fig. 3a). We found that sgRNAs targeting both motifs were significantly depleted in our peak-targeting CRISPRi screen (Fig. 3b). We reasoned that the p53 binding sites located within Peak 2319 are components of a downstream regulatory element that modulate RAD51C expression and selected sgRNAs targeting Peak 2319 and RAD51C for experimental validation.

Also among the most depleted sgRNAs in our peak-targeting CRISPRi screen were those targeting Peak 384 (Fig. 2h). In contrast to the close proximity between Peak 2319 and RAD51C, Peak 384 is located more than 200 kb away from the nearest annotated protein-coding gene (Fig. 3c). Peak 384 contains three p53 motifs, two of which were targeted by sgRNAs in our peak-targeting CRISPR library (Fig. 3c). We identified multiple sgRNAs targeting the first of those motifs that were significantly depleted in our peak-targeting CRISPRi screen (Fig. 3d). We hypothesized that the p53 binding sites within Peak 384 are components of a regulatory element located deep within an intergenic region of the genome and selected sgRNAs targeting this peak for experimental validation.

To experimentally validate that selected p53 binding sites represent functional regulatory elements we evaluated the impact of repressing each individual binding site on cell proliferation. We used lentivirus to stably transduce individual sgRNAs targeting the p53 binding sites of interest into dCas9-KRAB-expressing 769P cells. In addition, we generated stable dCas9-KRAB-expressing cell lines harboring an sgRNA targeting RAD51C, an sgRNA targeting an intergenic region of the genome, or an sgRNA with no genomic target. The resulting 7 cell lines were cultured in parallel for 18 days and population doublings were evaluated at each passage. Cell lines harboring sgRNAs targeting RAD51C, Peak 2319, and Peak 384 underwent significantly fewer population doublings as compared to cell lines containing negative control sgRNAs (Fig. 3e). Furthermore, we observed a significant difference in population doublings between cells harboring the sgRNA targeting the RAD51C TSS (RAD51C) and cells containing an sgRNA targeting the p53 binding site within the first intron of RAD51C (2319.1–1) (Fig. 3e). This observation suggests that sgRNAs targeting the RAD51C TSS and the RAD51C intron influence cell proliferation through distinct mechanisms (direct transcriptional interference of RAD51C and inhibition of regulatory element activity, respectively). We detected a similar impact on cell proliferation for two different sgRNAs targeting Peak 2319 in our validation experiments despite their differing degrees of depletion in our CRISPRi screen (Fig. 3b, e). This observation suggests that many of the modest proliferation phenotypes generated by sgRNAs in our CRISPRi screen may translate to more potent impacts on cell proliferation in focused validation experiments. Altogether, our results confirm that pooled CRISPR screens can be used to identify functional regulatory elements that influence cell proliferation.

In addition to the sgRNAs that were significantly depleted in our CRISPRi screen we identified several sgRNAs that were significantly enriched. For example, multiple sgRNAs targeting Peak 1267 resulted in a significant proliferative advantage in our CRISPRi screen (Fig. 2h). Peak 1267 contains five p53 motifs, two of which were targeted by sgRNAs in our peak-targeting CRISPR library (Additional file 3: Figure S3A). Although Peak 1267 is located within the first intron of TNFRSF10A, knockout of TNFRSF10A had no impact on cell proliferation in our gene-targeting CRISPRko screen (Additional file 3: Figure S3A**,** Figure S3B). In contrast, we identified multiple sgRNAs targeting the second p53 consensus motif in Peak 1267 that were significantly enriched in our peak-targeting CRISPRi screen (Additional file 3: Figure S3C). Importantly, these results demonstrate that regulatory elements can be functionally dissociated from proximal protein-coding genes.

### Pooled CRISPR screen identifies p53-bound regulatory elements that influence the DNA damage response

**Fig. 4:**
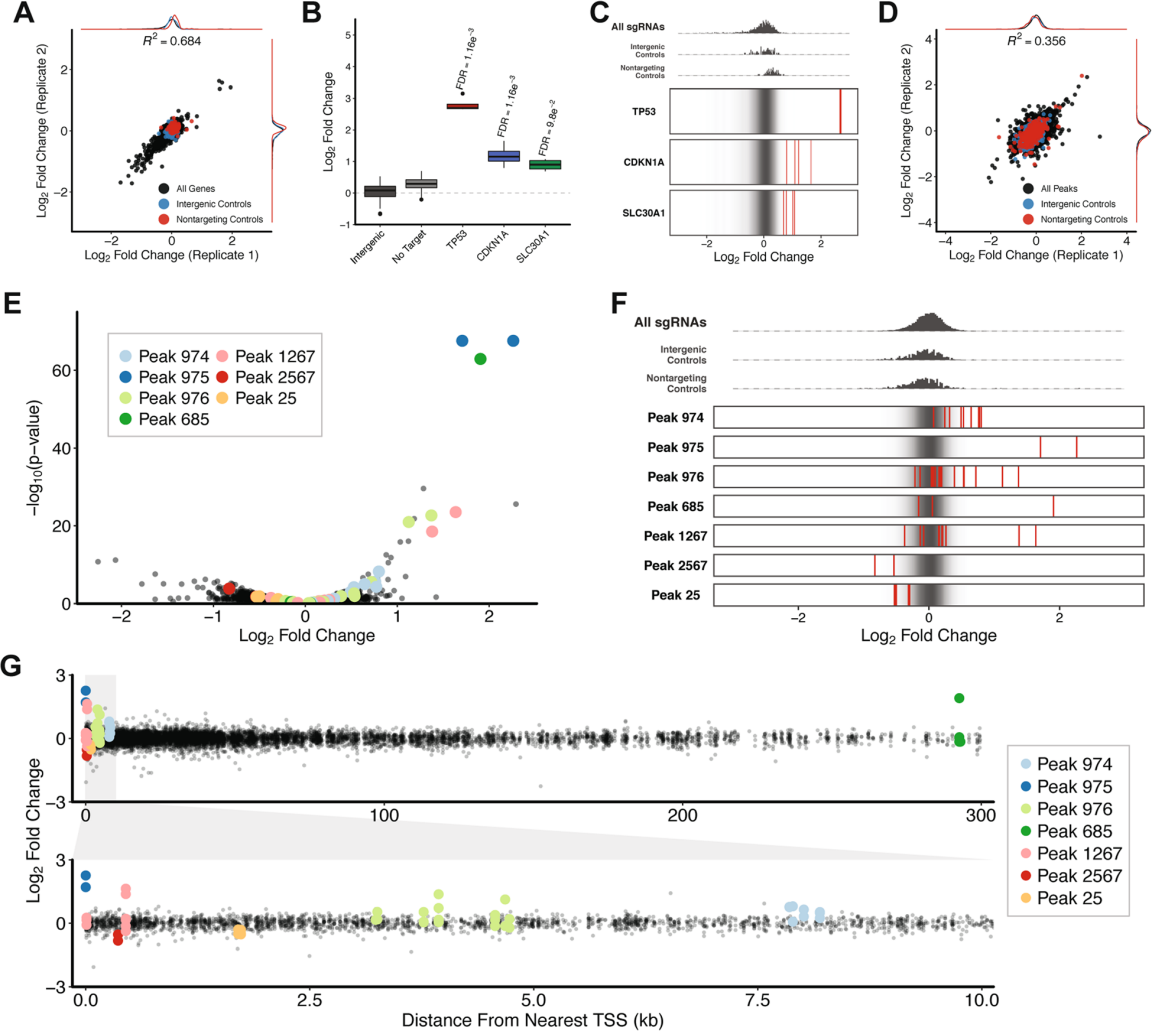
p53-bound regulatory elements influence cellular response to DNA damage. **a** Comparison of log_2_ fold changes (relative to pDNA) for all sgRNAs in gene-targeting CRISPR library between replicates. **b** Log_2_ fold changes (relative to pDNA) in CRISPR screen for sgRNAs targeting selected subset of genes. FDR values were calculated using the Benjamini-Hochberg method. **c** Visualization of enrichment/depletion for sgRNAs targeting a selected subset of genes (red) compared to all sgRNAs in CRISPR library (black). **d** Comparison of log_2_ fold changes (relative to pDNA) for all sgRNAs in peak-targeting CRISPR library between replicates. **e** Volcano plot comparing significance of sgRNA enrichment/depletion and log_2_ fold change (relative to pDNA) for all sgRNAs in CRISPR library. **f** Visualization of enrichment/depletion for sgRNAs targeting a selected subset of peaks (red) compared to all sgRNAs in CRISPR library (black). **g** Comparison of log_2_ fold change (relative to pDNA) and distance from nearest annotated TSS for all sgRNAs in CRISPR library

To evaluate the ability of a pooled CRISPR screen to identify regulatory elements that influence additional biological processes we next investigated the p53-mediated response to DNA damage. First, we utilized our gene-targeting CRISPR library to ensure that a CRISPR screen would be able to identify protein-coding genes that are required for cell cycle arrest in response to DNA damage. We infected Cas9-expressing 769P cells with our gene-targeting library at an MOI of ~ 0.5 and a representation of 1000 cells per sgRNA. Library-infected cells were cultured in the presence of the DNA damage-inducing agent doxorubicin for 21 days, genomic DNA was isolated, and targeted sequencing was performed to evaluate changes in sgRNA abundance relative to the CRISPR library pDNA (Additional file 8: Table S3). Analysis with MAGeCK revealed a strong correlation in sgRNA enrichment/depletion across biological replicates indicating that our screening results are highly reproducible (Fig. 4a, Additional file 18: Table S13). We identified several sgRNAs that prevented cell cycle arrest in response to DNA damage. (Fig. 4a, Additional file 18: Table S13). Among the most enriched sgRNAs were those targeting p53, CDKN1A, and SLC30A1 (Fig. 4b, Fig. 4c, Additional file 19: Table S14). These data demonstrate that a CRISPR screen can be used to identify genes that are required for cell cycle arrest in response to DNA damage.

We next used our peak-targeting CRISPRi library to search for regulatory elements involved in the p53-mediated response to DNA damage. We infected dCas9-KRAB-expressing 769P cells with our peak-targeting library at an MOI of ~ 0.5 and a representation of 1000 cells per sgRNA. Library-infected cells were cultured in the presence of doxorubicin for 21 days, genomic DNA was isolated, and targeted sequencing was performed to evaluate changes in sgRNA abundance relative to the CRISPR library pDNA (Additional file 12: Table S7). Analysis with MAGeCK revealed a relatively weak correlation in sgRNA enrichment/depletion across biological replicates (Fig. 4d). This weak correlation likely results from the combination of reduced proliferation in cells treated with doxorubicin and the less potent enrichments/depletions observed in screens performed with our peak-targeting CRISPR library. Despite weak overall correlation in sgRNA enrichment/depletion across replicates we were able to identify several sgRNAs that were significantly enriched in our screen (Fig. 4e, Additional file 20: Table S15). Interestingly, the three peaks that had the most significant impact on cycle arrest in response to DNA damage (Peak 974, Peak 975, and Peak 976) are located within a 15 kb window surrounding the CDKN1A transcription start site. Aside from p53, CDKN1A was the most enriched gene in our DNA damage screen performed with the gene-targeting CRISPR library (Fig. 4b, c, f, Additional file 21: Table S16). Although most of the p53 binding sites identified in our screen were located within 10 kb of an annotated TSS, at least one was located more than 250 kb away from the nearest TSS (Fig. 4g). Altogether, these data provide an additional example of a pooled CRISPR screen being used to successfully identify functional regulatory elements.

We again tested the ability of CRISPRko technology to identify functional regulatory elements by performing a DNA damage response screen in cells expressing Cas9 as opposed to dCas9-KRAB. We infected Cas9-expressing 769P cells with our peak-targeting library at an MOI of ~ 0.5 and a representation of 1000 cells per sgRNA. Library-infected cells were cultured in the presence of doxorubicin for 21 days, genomic DNA was isolated, and targeted sequencing was performed to evaluate changes in sgRNA abundance relative to the CRISPR library pDNA (Additional file 15: Table S10). Analysis with MAGeCK revealed a moderate correlation in sgRNA enrichment/depletion across biological replicates (Additional file 4: Figure S4A, Additional file 22: Table S17). While we did identify p53 binding sites in which CRISPR-mediated knockout prevented cell cycle arrest in response to DNA damage, the magnitude of sgRNA enrichment was less significant as compared to the CRISPRi screen (Additional file 4: Figure S4B, Additional file 23: Table S18). Moreover, the sgRNA enrichments were far less pronounced than we observed in the CRISPRi screen (Additional file 4: Figure S4C, Figure S4D). Once again, we observed minimal overlap in the sgRNAs that were significantly enriched/depleted across the CRISPRko and CRISPRi screens of the DNA damage response (Additional file 4: Figure S4E). Furthermore, none of the p53 binding sites that appeared to impact the DNA damage response in the CRISPRko were located near genes that were significantly enriched/depleted in our gene-targeting CRISPR screen. Based on these data we focused our validation efforts on p53 binding sites identified in our CRISPRi screen.

### Repression of p53-bound regulatory elements prevents cell cycle arrest in response to DNA damage

**Fig. 5:**
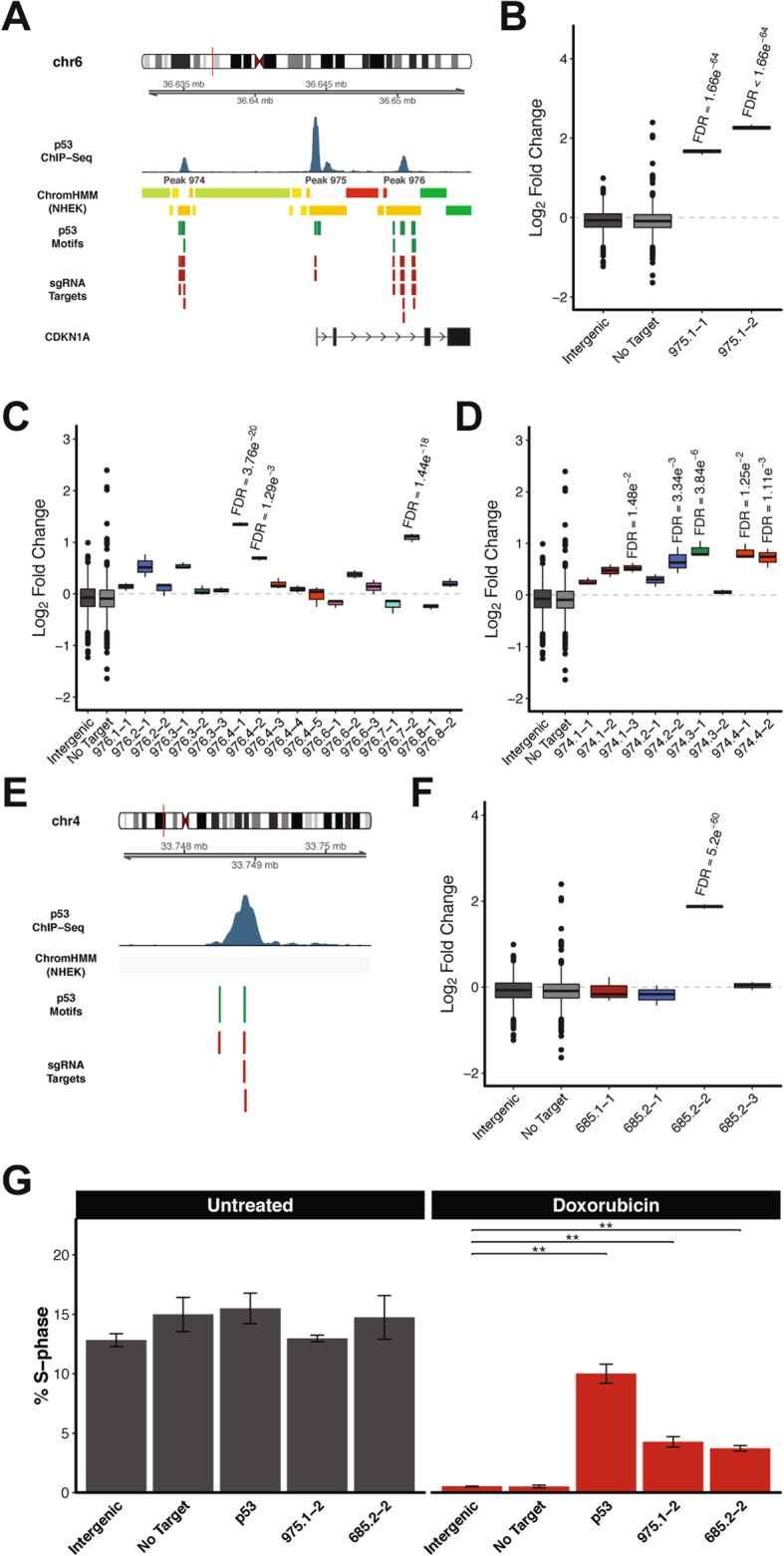
Functional characterization of p53-bound regulatory elements that influence cellular response to DNA damage. **a** Schematic of p53 motifs and sgRNA targets located in Peaks 974, 975, and 976. (ChromHMM track legend: red = active promoter; orange = strong enhancer; yellow = weak/poised enhancer; dark green = transcriptional transition/elongation; light green = weak transcribed) (**b-d**) Log_2_ fold changes (relative to pDNA) in CRISPR screen for sgRNAs targeting **b** Peak 975, **c** Peak 976, and **d** Peak 974. FDR values were calculated using the Benjamini-Hochberg method. **e** Schematic of p53 motifs and sgRNA targets located in Peak 685. **f** Log_2_ fold changes (relative to pDNA) in CRISPR screen for sgRNAs targeting Peak 685. FDR values were calculated using the Benjamini-Hochberg method. **g** Cell cycle analysis of DNA damage response following inhibition of Peak 975 or Peak 685. *P*-values were calculated using the two-tailed unpaired Student’s t-test with equal variances. **P < 0.01

Among the sgRNAs that were most enriched in our peak-targeting CRISPRi screen of the DNA damage response were those targeting ChIP-Seq peaks nearest CDKN1A (Fig. 4f). More specifically, Peak 975 overlaps the CDKN1A TSS, Peak 974 is located 10 kb upstream of the CDKN1A TSS, and Peak 976 is located 5 kb downstream of the CDKN1A TSS (Fig. 5a). Peak 975 contains three p53 consensus motifs and multiple sgRNAs targeting the first of those motifs were significantly enriched in our CRISPRi screen (Fig. 5b). Peak 976 contains eight p53 consensus motifs and we identified sgRNAs targeting several of those motifs that were significantly enriched in our CRISPRi screen (Fig. 5c). Lastly, Peak 974 contains four p53 consensus motifs and sgRNAs targeting each of those motifs were significantly enriched in our CRISPRi screen, although the magnitude of enrichment was not as pronounced as with sgRNAs targeting Peak 975 and Peak 976 (Fig. 5d). We hypothesized that the p53 binding sites located within these ChIP-Seq peaks are components of regulatory elements that modulate CDKN1A expression and selected an sgRNA targeting Peak 975 for experimental validation.

Also among the most enriched sgRNAs in our peak-targeting CRISPRi screen of the DNA damage response was one targeting Peak 685 (Fig. 4e). Peak 685 is located more than 250 kb away from the nearest annotated protein-coding gene and contains two p53 motifs, both of which were targeted by sgRNAs in our peak-targeting CRISPR library (Fig. 5e). We identified one sgRNA targeting the second of those motifs that was significantly enriched in our peak-targeting CRISPRi screen (Fig. 5f). We hypothesized that this p53 binding site is a component of a regulatory element located deep within an intergenic region of the genome and selected an sgRNA targeting Peak 685 for experimental validation.

To experimentally validate that the selected p53 binding sites represent functional regulatory elements we evaluated the impact of repressing individual binding sites on cell cycle arrest in response to DNA damage. We used lentivirus to stably transduce individual sgRNAs targeting p53 binding sites of interest into dCas9-KRAB-expressing 769P cells. In addition, we generated stable dCas9-KRAB-expressing cell lines harboring an sgRNA targeting p53, an sgRNA targeting an intergenic region of the genome, or an sgRNA with no genomic target. The resulting 5 cell lines were cultured in the presence or absence of doxorubicin for 16 h followed by cell cycle analysis (Additional file 5: Figure S5). All of the stable cell lines we generated displayed similar cell cycle profiles in standard culture conditions, with 12–15% of total cells in S-phase (Fig. 5g). In response to doxorubicin treatment cell lines harboring negative control sgRNAs dropped to 0.5% of total cells in S-phase (Fig. 5g). In contrast, 10% of cells harboring sgRNAs targeting p53 remained in S-phase after treatment with doxorubicin (Fig. 5g). Likewise, cells containing sgRNAs targeting Peak 975 and Peak 685 exhibited significantly lower levels of cell cycle arrest with 4.27 and 3.72% of total cells in S-phase, respectively (Fig. 5g). Altogether, these results confirm that pooled CRISPR screens can be used to identify functional regulatory elements that influence the DNA damage response. Moreover, these data further demonstrate that pooled CRISPR screening can be used as a general approach to identify functional regulatory elements that influence diverse biological processes.

## Discussion

In recent years CRISPR-based approaches have emerged as powerful tools for functional genomics studies. Pooled CRISPR screens in particular have been widely used to identify protein-coding genes involved in a variety of cellular pathways and processes [5–8]. Here, we explore the application of pooled CRISPR screening in the functional characterization of regulatory elements throughout the genome. We developed a cell-based screening system to profile the functional role of p53-bound regulatory elements in multiple biological contexts. Using this system we identified several regulatory elements that influence cell proliferation and/or cell cycle arrest in response to DNA damage. Moreover, the functional significance for several of the regulatory elements we uncovered was confirmed using orthogonal experimental approaches.

The regulatory elements identified in our screens varied greatly in their proximity to the genes in which they regulate. Among the most conventional regulatory elements we identified were those within Peak 2319 and Peak 975. These peaks are both located within 1 kb of a transcription start site (RAD51C and CDKN1A, respectively) and their inhibition phenocopied the effect of gene knockout. Surprisingly, some of the regulatory elements identified in our screens were in close proximity to transcription start sites but were not functionally associated with nearby genes. For example, inhibition of Peak 1267 had a significant impact on both cell proliferation and cell cycle arrest in response to DNA damage. Although Peak 1267 is located within 1 kb of the TNFRSF10A transcription start site, knockout of TNFRSF10A had no effect on either process. Despite its proximity to TNFRSF10A, Peak 1267 likely functions as a distal regulatory element for other genes involved in cell proliferation and/or the DNA damage response.

Several of the regulatory elements we discovered are located in intergenic regions of the genome that are devoid of any prior functional annotation. For example, Peak 384 is located more than 200 kb away from the nearest annotated gene yet inhibition of this site resulted in a significant reduction in cell proliferation. Among the p53 binding sites that had the greatest impact on cell cycle arrest in response to DNA damage was Peak 685. Interestingly, this peak is located more than 250 kb from the nearest annotated gene. These examples highlight the ability of pooled CRISPR screens to identify functional regulatory elements located deep within intergenic regions the human genome. While we hypothesize that the activity of these intergenic regulatory elements is p53-dependent, we cannot exclude the possibility that they influence cell proliferation and/or cell cycle arrest through a p53-independent mechanism. Regardless, identifying the gene targets of these noncoding regulatory elements will be an exciting area of future research.

In the course of our study we evaluated multiple methods for perturbing the function of regulatory elements. More specifically, we performed CRISPRi screens with cells expressing dCas9-KRAB as well as CRISPRko screens with cells expressing Cas9. While pooled CRISPR screens have been performed using dCas9 in the absence of a KRAB domain fusion, we reasoned that the repressive properties of dCas9-KRAB would result in greater impacts on regulatory element function and, in turn, more pronounced cellular phenotypes in our screens. Both CRISPRi and CRISPRko screening methods resulted in the enrichment/depletion of sgRNAs targeting p53 binding sites, although we observed minimal overlap between the different approaches. We identified only one p53 binding site (Peak 1267) targeted by sgRNAs that were enriched using both technologies. Importantly, the sgRNA enrichments/depletions we observed in our peak-targeting CRISPRi screens more closely reflected the results of our gene-targeting CRISPRko screens. We hypothesize that the degenerate nature of the p53 consensus motif allows p53 binding sites to be tolerant of indels that result from DNA cleavage by Cas9 and prevents CRISPRko-based approaches from effectively perturbing their function. Given that many transcription factors bind to degenerate sequence motifs we recommend the use of CRISPRi-based methods when performing pooled CRISPR screens focused on the identification of functional regulatory elements. In contrast, CRISPRko-based approaches that introduce indels would be more appropriate for screens intended to functionally profile the sequences of known regulatory elements at high resolution. Likewise, CRISPR screens performed using dCas9 (without a KRAB domain fusion) could interfere with transcription factor binding and distinguish sites within a regulatory element that are required for activity.

With regards to our CRISPR library design, we chose to target all PAM-containing sequences that were proximal to p53 consensus motifs within p53 binding sites when constructing our peak-targeting CRISPR library. While sgRNA design principles have been described for both CRISPRi and CRISPRko approaches, the methods for generating these rules have been based exclusively on the perturbation of protein-coding gene function [14, 35, 36]. Consequently, many of the features that are incorporated into these design rules (e.g. location within coding sequence, distance to TSS) are not applicable to the design of sgRNAs targeting regulatory elements. The inclusion of all possible sgRNAs in our peak-targeting CRISPR library did result in more variability between sgRNAs with common targets than is typically observed in gene-targeting CRISPR screens performed with optimized sgRNA libraries. However, our comprehensive library design was essential for the identification of functional regulatory elements in this study. Some of the p53 motifs profiled in our screens could only be targeted by one sgRNA, which raises the concern that the resulting phenotypes might be caused by off-target effects. However, many of those motifs occurred within p53 ChIP-Seq peaks that harbored multiple p53 motifs and were targeted by several additional sgRNAs. Future efforts to characterize sgRNA design rules when targeting regulatory elements will aid in distinguishing active from inactive sgRNAs, reduce the potential for off-target effects, and lead to increased reproducibility across candidate sgRNAs with common targets. Moreover, optimized sgRNA design principles would reduce the number of sgRNAs required to effectively screen regulatory elements and ultimately increase the number of genomic regions that can be profiled in a pooled CRISPR screen.

In this study we developed screening systems to identify p53-bound regulatory elements that influence cell proliferation and/or cell cycle arrest in response to DNA damage. However, the approaches we describe here can be adapted to profile the function of regulatory elements bound by any transcription factor. Alternatively, these methods can be utilized to interrogate the function of intergenic regions of interest identified in genome-wide association studies (GWAS) or noncoding regions that have been shown to harbor rare genetic variants identified in patient samples. One major challenge associated with each of the aforementioned applications is the development of relevant cell-based model systems in which to perform pooled CRISPR screens.

## Conclusions

In conclusion, our findings demonstrate that CRISPR-based screening methods can be used to characterize the functional role of noncoding regulatory elements in diverse biological processes. Moreover, our results provide valuable insight into practical considerations for the design and implementation of CRISPR screens focused on noncoding regions of the genome.

## Methods

### Cell culture

Human renal adenocarcinoma cells (769P, ATCC CRL-1933) were cultured in RPMI medium supplemented with 10% Fetal Bovine Serum (Sigma-Aldrich). Cells were cultured with 250 nM doxorubicin (Sigma) for pooled CRISPR screens and validation experiments to induce DNA damage. Cells were tested periodically for mycoplasma contamination and were authenticated by SNP fingerprinting at both the initiation and completion of each CRISPR screen.

### Generation of Cas9-expressing cell lines

Lentivirus was produced from vectors encoding Cas9 (Addgene, 96924) and dCas9-KRAB (Addgene, 96918) as previously described [14]. For infection, 1.5e6 cells were plated in a 12-well tissue culture dish along with 2 mL media, 700 μL lentivirus, and 1 mg/mL polybrene per well. Plates were then centrifuged at 930 xg for 2 h at 30 °C. Following centrifugation 2 mL of fresh media was added dropwise to each well and cells were incubated overnight at 37 °C. Cells were collected 24 h post-infection and transferred into flasks with fresh media supplemented with 4 μg/mL blasticidin. Cells were cultured in the presence of blasticidin throughout expansion and Cas9 expression was confirmed by western blot.

### Western blot

Cell pellets were lysed and protein concentrations were quantified by BCA assay (ThermoFisher). Western blots were performed on protein lysates (60 μg/well). Primary antibodies used were anti-Cas9 (Cell Signaling Technology, 14697) and anti-H3 (Cell Signaling Technology, 14269). Protein was visualized using an IRDye800CW-conjugated secondary antibody (Licor, 926–32210).

### Gene-targeting CRISPR library design and construction

The gene-targeting CRISPR library was designed against 330 protein-coding genes that are predicted targets of p53 [29]. The library was comprised of 4 sgRNAs against each p53 target gene as well as 4 sgRNAs against p53. As controls, 70 sgRNAs targeting intergenic sites and 70 sgRNAs with no genomic target were included in the library. All sgRNA sequences are listed in Additional file 7: Table S2. Library cloning into lentiGuide-Puro (Addgene, 52963) and large-scale virus production was performed as previously described [14].

### Peak-targeting CRISPR library design and construction

The peak-targeting CRISPR library was designed against 2036 previously characterized p53 ChIP-Seq peaks [29]. The library was comprised of sgRNAs targeting all possible PAM-containing sites located within 16 bp of a p53 consensus motif (CWWG [N]_2-12_CWWG). All sgRNAs with putative off-target sites within protein-coding genes were excluded from the library. In addition, sgRNAs with more than one potential off-target site within unrelated noncoding regions of the genome were excluded. Cutting frequency determination (CFD) was used to evaluate off-target potential with a CFD = 1 being considered a potential off-target site [36]. As controls, 500 sgRNAs targeting intergenic sites and 500 sgRNAs with no genomic target were included in the library. All sgRNA sequences are listed in Additional file 11: Table S6. Library cloning into lentiGuide-Puro (Addgene, 52963) and large-scale virus production was performed as previously described [14].

### Pooled CRISPR screens

The lentiviral titer for each pooled CRISPR library was determined as previously described [14]. Library transductions were performed with an estimated transduction efficiency of 30–50% using sufficient cell numbers such that 1000 cells/sgRNA remained after puromycin selection. Cells were cultured in the presence of puromycin (6 μg/mL) for 7 days post-infection to ensure complete selection. At each passage adequate cell numbers were re-seeded to maintain a library representation of 1000 cells/sgRNA. At the completion of each screen cell pellets were collected, washed with PBS, and frozen at − 20 °C prior to gDNA isolation. All screens were performed in biological triplicate.

### Targeted sequencing libraries

Genomic DNA was isolated using NucleoSpin Blood XL, Blood L, and Blood columns according to the manufacturer’s protocol (Machery Nagel). Targeted sequencing libraries were generated directly from genomic DNA as previously described [14]. Libraries were size-selected using Agencourt AMPure XP beads (Beckman Coulter). Purified libraries were sequenced on a HiSeq 2500 (Illumina).

### Pooled CRISPR screen analysis

Sequencing reads were translated into read counts by extracting the 20 nt sgRNA sequence from each read followed by alignment to the library reference. For each screen we achieved an average sequencing depth of > 500 aligned reads per sgRNA per replicate. Enrichment/depletion of sgRNAs in each screen was determined with MAGeCK using the alphamedian option to calculate fold changes [34]. Fold changes in sgRNA abundances were calculated relative to their abundance in the initial plasmid DNA library.

### Generation of stable CRISPRi cell lines

Individual sgRNAs were cloned into lentiGuide-Puro (Addgene, 52963) and lentivirus was generated as previously described [14]. For infection, 1.5e6 dCas9-KRAB-expressing cells were plated in a 12-well tissue culture dish along with 2 mL media, 150 μL lentivirus, and 1 mg/mL polybrene per well. Plates were then centrifuged at 930 xg for 2 h at 30 °C. Following centrifugation 2 mL of fresh media was added dropwise to each well and cells were incubated overnight at 37 °C. Cells were collected 24 h post-infection and transferred into flasks with fresh media supplemented with 4 μg/mL blasticidin and 6 μg/mL puromycin. Cells were cultured in the presence of blasticidin and puromycin throughout expansion. The sgRNA sequences used to generate each cell line are listed in Additional file 24: Table S19.

### Cell proliferation analysis

Stable CRISPRi cell lines harboring individual sgRNAs were seeded at equal cell numbers and cultured in parallel for 18 days. Every 2–3 days cells were collected, counted, and re-seeded at equal cell numbers. All experiments were performed in triplicate.

### Cell cycle analysis

Stable CRISPRi cell lines harboring individual sgRNAs were seeded at equal cell numbers and cultured in the presence or absence of doxorubicin. Cell cycle analysis was performed using the 488 EdU Click Proliferation Kit (BD Biosciences, 565455) in conjunction with propidium iodide staining as per the manufacturer’s guidelines. All experiments were performed in triplicate.

## Supplementary information

**Additional file 1: Figure S1.** p53 enrichment in pooled CRISPR screens requires wildtype p53. p53 enrichment scores (categorized by p53 mutation status) from pooled CRISPR knockout screens in 350 cancer cell lines. *P*-values were calculated using the two-tailed unpaired Student’s t-test with equal variances. ***P < 0.001, **P < 0.01.

**Additional file 2:** **Figure S2.** CRISPR-knockout screen identifies p53-bound regulatory elements that influence cell proliferation. (**A**) Comparison of log_2_ fold changes (relative to pDNA) for all sgRNAs between replicates in 769P-Cas9 cells. (**B**) Volcano plot comparing significance of sgRNA enrichment/depletion and log_2_ fold change (relative to pDNA) in 769P-Cas9 cells for all sgRNAs in CRISPR library. (**C**) Visualization of enrichment/depletion in 769P-Cas9 cells for sgRNAs targeting a selected subset of peaks (red) compared to all sgRNAs in CRISPR library (black). (**D**) Comparison of log_2_ fold change (relative to pDNA) and distance from nearest annotated TSS for all sgRNAs in CRISPR library. (**E**) Comparison of log_2_ fold changes (relative to pDNA) for all sgRNAs between 769P-Cas9 and 769P-dCas9-KRAB screens.

**Additional file 3:** **Figure S3.** CRISPR screens uncover functional dissociation of p53-bound regulatory element and proximal protein-coding gene. (**A**) Schematic of p53 motifs and sgRNA targets located in Peak 1267. (ChromHMM track legend: red = active promoter) (**B**) Log_2_ fold changes (relative to pDNA) in CRISPR screen for sgRNAs targeting TNFRSF10A in 769P-Cas9 cells. (**C**) Log_2_ fold changes (relative to pDNA) in CRISPR screen for sgRNAs targeting Peak 1267 in 769P-dCas9-KRAB cells. FDR values were calculated using the Benjamini-Hochberg method.

**Additional file 4: Figure S4.** CRISPR-knockout screen identifies p53-bound regulatory elements that influence cellular response to DNA damage. (**A**) Comparison of log_2_ fold changes (relative to pDNA) for all sgRNAs between replicates in 769P-Cas9 cells. (**B**) Volcano plot comparing significance of sgRNA enrichment/depletion and log_2_ fold change (relative to pDNA) in 769P-Cas9 cells for all sgRNAs in CRISPR library. (**C**) Visualization of enrichment/depletion in 769P-Cas9 cells for sgRNAs targeting a selected subset of peaks (red) compared to all sgRNAs in CRISPR library (black). (**D**) Comparison of log_2_ fold change (relative to pDNA) and distance from nearest annotated TSS for all sgRNAs in CRISPR library. (**E**) Comparison of log_2_ fold changes (relative to pDNA) for all sgRNAs between 769P-Cas9 and 769P-dCas9-KRAB screens.

**Additional file 5:** **Figure S5.** p53 inhibition influences cellular response to DNA damage. Raw flow cytometry data from cell cycle analysis experiments.

**Additional file 6:** **Table S1.** Project Achilles – p53 status and enrichment data.

**Additional file 7: Table S2.** CRISPR library targeting p53-regulated protein-coding genes.

**Additional file 8:** **Table S3.** CRISPR screen raw read counts (Gene Library/Cas9).

**Additional file 9:** **Table S4.** Guide-level MAGeCK output (Gene Library/Cas9/Untreated).

**Additional file 10:** **Table S5.** Gene-level MAGeCK output (Gene Library/Cas9/Untreated).

**Additional file 11:** **Table S6.** CRISPR library targeting p53 binding sites.

**Additional file 12:** **Table S7.** CRISPR screen raw read counts (Peak Library/dCas9-KRAB).

**Additional file 13:** **Table S8.** Guide-level MAGeCK output (Peak Library/dCas9-KRAB/Untreated).

**Additional file 14: Table S9.** Peak-level MAGeCK output (Peak Library/dCas9-KRAB/Untreated).

**Additional file 15:Table S10.** CRISPR screen raw read counts (Peak Library/Cas9).

**Additional file 16:** **Table S11.** Guide-level MAGeCK output (Peak Library/Cas9/Untreated).

**Additional file 17: Table S12.** Peak-level MAGeCK output (Peak Library/Cas9/Untreated).

**Additional file 18: Table S13.** Guide-level MAGeCK output (Gene Library/Cas9/ Doxorubicin).

**Additional file 19: Table S14.** Gene-level MAGeCK output (Gene Library/Cas9/Doxorubicin).

**Additional file 20: Table S15.** Guide-level MAGeCK output (Peak Library/dCas9-KRAB/ Doxorubicin).

**Additional file 21:** **Table S16.** Peak-level MAGeCK output (Peak Library/dCas9-KRAB/Doxorubicin)

**Additional file 22:** **Table S17.** Guide-level MAGeCK output (Peak Library/Cas9/ Doxorubicin).

**Additional file 23: Table S18.** Peak-level MAGeCK output (Peak Library/Cas9/ Doxorubicin).

**Additional file 24: Table S19.** Sequences of sgRNAs used in validation experiments.

## Data Availability

The Gene Expression Omnibus accession number for the pooled CRISPR screens described in this paper is GSE126320.

## Abbreviations

CFD: Cutting frequency determination
CRISPR: Clustered regularly interspaced short palindromic repeats
CRISPRi: CRISPR interference
CRISPRko: CRISPR knockout
MAGeCK: Model-based analysis of genome-wide CRISPR-Cas9 knockout
MOI: Multiplicity of infection
sgRNA: single guide RNA
TSS: Transcription start site
