# Supplementary figures and images for: Identification of functional regulatory elements in the human genome using pooled CRISPR screens
Source: BMC Genomics. 2020 Jan 31;21:107. doi: 10.1186/s12864-020-6497-0 (PMC6995077; doi:10.1186/s12864-020-6497-0)

# Supplemental Figure S1

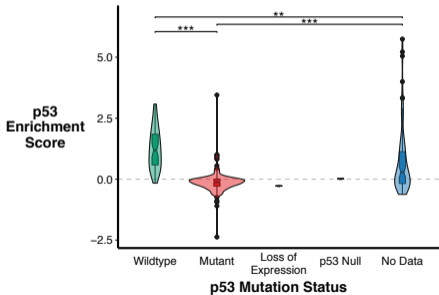

Supplement: Supplementary file 1 — Additional file 1: Figure S1. p53 enrichment in pooled CRISPR screens requires wildtype p53. p53 enrichment scores (categorized by p53 mutation status) from pooled CRISPR knockout screens in 350 cancer cell lines. P-values were calculated using the two-tailed unpaired Student’s t-test with equal variances. ***P < 0.001, **P < 0.01. [file 12864_2020_6497_MOESM1_ESM.pdf]

# Supplemental Figure S2

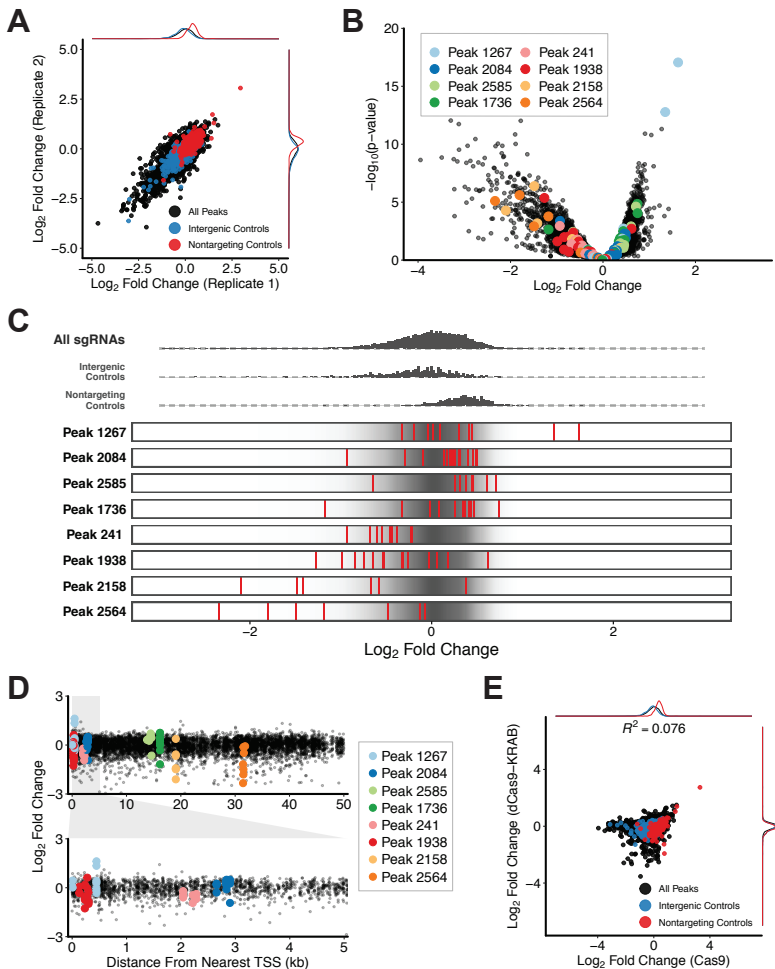

Supplement: Supplementary file 2 — Additional file 2: Figure S2. CRISPR-knockout screen identifies p53-bound regulatory elements that influence cell proliferation. (A) Comparison of log2 fold changes (relative to pDNA) for all sgRNAs between replicates in 769P-Cas9 cells. (B) Volcano plot comparing significance of sgRNA enrichment/depletion and log2 fold change (relative to pDNA) in 769P-Cas9 cells for all sgRNAs in CRISPR library. (C) Visualization of enrichment/depletion in 769P-Cas9 cells for sgRNAs targeting a selected subset of peaks (red) compared to all sgRNAs in CRISPR library (black). (D) Comparison of log2 fold change (relative to pDNA) and distance from nearest annotated TSS for all sgRNAs in CRISPR library. (E) Comparison of log2 fold changes (relative to pDNA) for all sgRNAs between 769P-Cas9 and 769P-dCas9-KRAB screens. [file 12864_2020_6497_MOESM2_ESM.pdf]

# Supplemental Figure S3

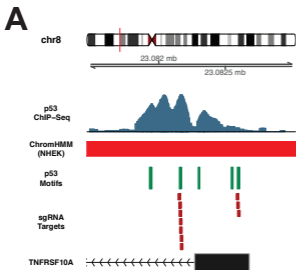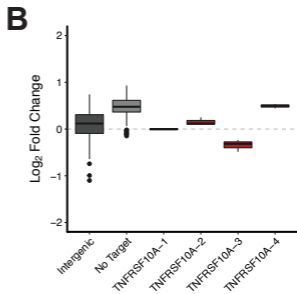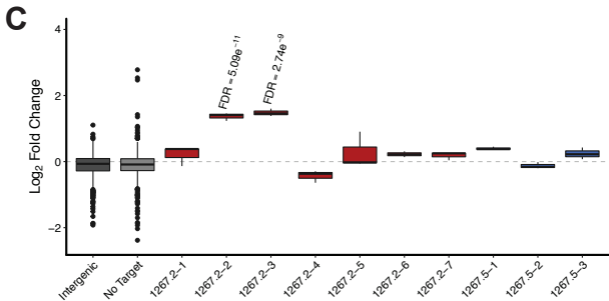

Supplement: Supplementary file 3 — Additional file 3: Figure S3. CRISPR screens uncover functional dissociation of p53-bound regulatory element and proximal protein-coding gene. (A) Schematic of p53 motifs and sgRNA targets located in Peak 1267. (ChromHMM track legend: red = active promoter) (B) Log2 fold changes (relative to pDNA) in CRISPR screen for sgRNAs targeting TNFRSF10A in 769P-Cas9 cells. (C) Log2 fold changes (relative to pDNA) in CRISPR screen for sgRNAs targeting Peak 1267 in 769P-dCas9-KRAB cells. FDR values were calculated using the Benjamini-Hochberg method. [file 12864_2020_6497_MOESM3_ESM.pdf]

# Supplemental Figure S4

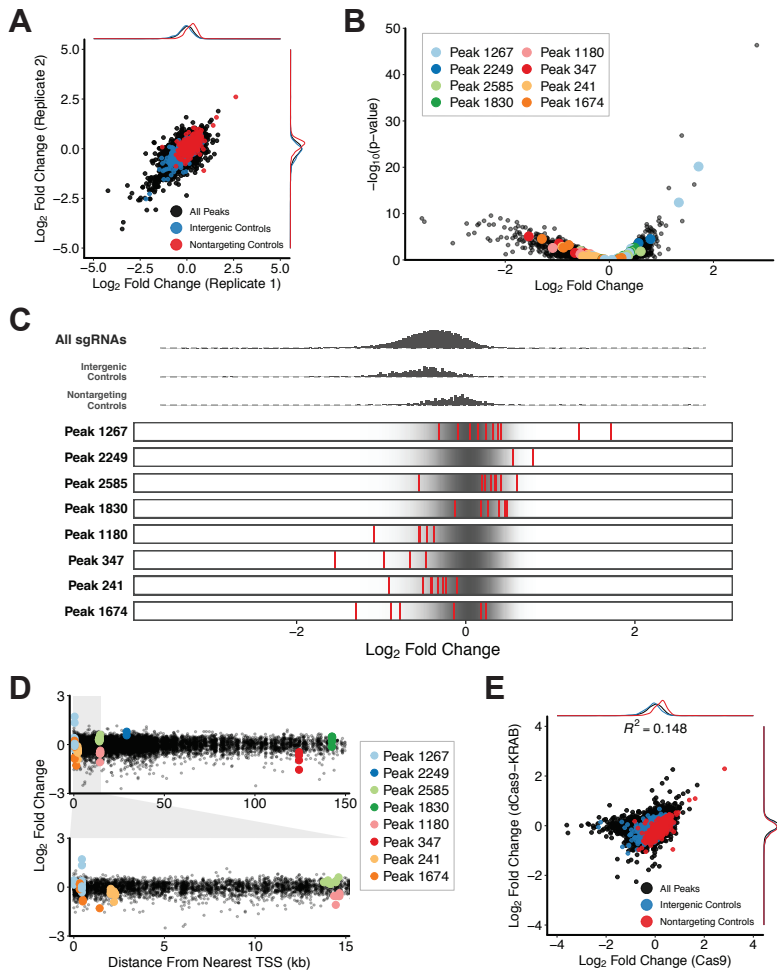

Supplement: Supplementary file 4 — Additional file 4: Figure S4. CRISPR-knockout screen identifies p53-bound regulatory elements that influence cellular response to DNA damage. (A) Comparison of log2 fold changes (relative to pDNA) for all sgRNAs between replicates in 769P-Cas9 cells. (B) Volcano plot comparing significance of sgRNA enrichment/depletion and log2 fold change (relative to pDNA) in 769P-Cas9 cells for all sgRNAs in CRISPR library. (C) Visualization of enrichment/depletion in 769P-Cas9 cells for sgRNAs targeting a selected subset of peaks (red) compared to all sgRNAs in CRISPR library (black). (D) Comparison of log2 fold change (relative to pDNA) and distance from nearest annotated TSS for all sgRNAs in CRISPR library. (E) Comparison of log2 fold changes (relative to pDNA) for all sgRNAs between 769P-Cas9 and 769P-dCas9-KRAB screens. [file 12864_2020_6497_MOESM4_ESM.pdf]

Supplemental Figure S5

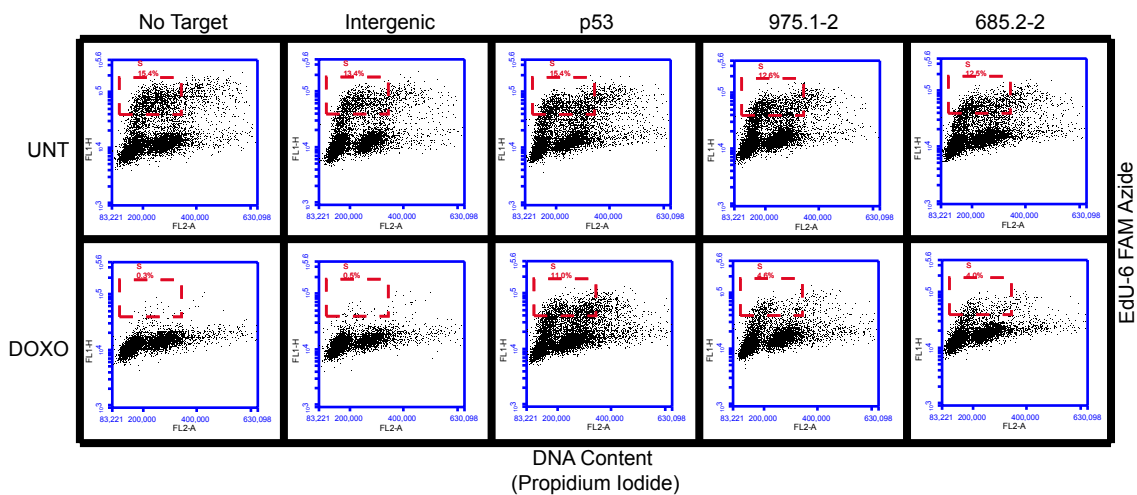

Supplement: Supplementary file 5 — Additional file 5: Figure S5. p53 inhibition influences cellular response to DNA damage. Raw flow cytometry data from cell cycle analysis experiments. [file 12864_2020_6497_MOESM5_ESM.pdf]
